# Isoalantolactone suppresses gallbladder cancer progression via inhibiting the ERK signalling pathway

**DOI:** 10.1080/13880209.2023.2191645

**Published:** 2023-03-30

**Authors:** Xingyu Lv, Yuqi Lin, Xi Zhu, Xiujun Cai

**Affiliations:** Department of General Surgery, Sir Run Run Shaw Hospital, Zhejiang University School of Medicine, Hangzhou, China

**Keywords:** IAL, traditional Chinese medicine, antitumour drug, Ro 67-7476, MAPK

## Abstract

**Context:**

Gallbladder cancer (GBC) is the most common malignant tumour of the biliary tract. Isoalantolactone (IAL), an active sesquiterpene lactone compound isolated from the roots of *Inula helenium* L. (Asteraceae), has antitumour effects.

**Objective:**

This study investigates the effects of IAL on GBC.

**Materials and methods:**

*In vitro*, NOZ and GBC-SD cells were treated with IAL (0, 10, 20 and 40 μM) for 24 h. The DMSO-treated cells were selected as a control. Cell proliferation, migration, invasion and apoptosis were measured by the CCK-8 assay, transwell assay, flow cytometry and western blot. *In vivo*, subcutaneous tumour xenografts were constructed by injecting nude mice (BALB/C) with 5 × 10^6^ NOZ cells. Mice were divided into the control group (equal amount of DMSO), the IAL group (10 mg/kg/day) and the IAL + Ro 67-7476 group (IAL, 10 mg/kg/day; Ro 67-7476, 4 mg/kg/day). The study duration was 30 days.

**Results:**

Compared with the DMSO group, cell proliferation of NOZ (IC_50_ 15.98 μM) and GBC-SD (IC_50_ 20.22 μM) was inhibited by about 70% in the IAL 40 μM group. Migration and invasion were suppressed by about 80%. Cell apoptosis rate was increased about three-fold. The phosphorylation level of ERK was decreased to 30–35%. Tumour volume and weight (about 80% reduction) were suppressed by IAL *in vivo*. Moreover, the effects of IAL were abolished by Ro 67-7476 *in vitro* and *in vivo*.

**Discussion and conclusions:**

Our findings indicate that IAL could inhibit GBC progression *in vitro* and *in vivo* by inhibiting the ERK signalling pathway.

## Introduction

Gallbladder cancer (GBC), the most common biliary tract cancer, is an aggressive malignant tumour derived from epithelial cells of the gallbladder (Mao et al. 2020). Due to rapid tumour progression and the lack of early diagnosis, GBC is often diagnosed at the late stage with a high incidence of liver metastasis (Hundal and Shaffer 2014). Although surgical resection is currently the most effective strategy for treating GBC, less than 30% of GBC patients could receive surgical resection (Baiu and Visser 2018). Most GBC patients with distant metastasis lose the opportunity of radical surgery and only receive non-surgical treatment such as chemotherapy and radiotherapy (Song et al. 2020). However, the existing gemcitabine-based conventional chemotherapy often results in severe systemic toxicity and drug resistance in GBC patients (Yang et al. 2019). Therefore, finding a novel potential drug and identifying its underlying mechanism of activity are urgently needed to improve treatment outcomes for GBC patients.

Chinese herbal medicine, a source of broad pharmaceutical potential, is widely used in the treatment of various diseases and has been considered as one of the most important sources of antitumour drugs (Li and Weng 2017). Isoalantolactone (IAL) is an active sesquiterpene lactone compound isolated from the roots of *Inula helenium* L. (Asteraceae), exhibits various biological and pharmacological effects such as antitumour, anti-inflammatory and antioxidant (Seo et al. 2017; Ding et al. 2019; Lu et al. 2020). Increasing evidence suggests that IAL had a highly selective cytotoxic effect on cancer cells, but a low toxicity on normal cells (Chen et al. 2018). The pleiotropic antitumour effects of IAL have been demonstrated in a variety of malignancies including hepatocellular carcinoma, breast carcinoma, prostate adenocarcinoma, oesophagus cancer, ovarian carcinoma and pancreatic carcinoma (Khan et al. 2012; Li et al. 2016; Weng et al. 2016; Chen et al. 2018; Lu et al. 2018; Kim et al. 2021). However, the therapeutic effect of IAL on GBC and its underlying molecular mechanism remain unrevealed. Based on its significant inhibitory effect on other malignant tumours, we hypothesized that IAL could also have a therapeutic effect on GBC. In this study, we found that IAL could suppress GBC progression through ERK signalling pathway. Meanwhile, our study provided a theoretical basis for the clinical application of IAL in the treatment of GBC.

## Materials and methods

### Reagents and cell culture

Isoalantolactone (HY-N0780) and Ro 67-7476 (HY-100403) were obtained from MedChemExpress (MCE, South Brunswick Township, NJ). The human gallbladder carcinoma cell lines, including NOZ, GBC-SD, EH-GB1 and SGC-996, and the normal human intrahepatic biliary epithelial cells (HiBECs) were purchased from the Cell Bank of the Chinese Academy of Sciences (Shanghai, China). The cells were cultured in Dulbecco’s modified Eagle medium (DMEM, Gibco, Carlsbad, CA) supplemented with 10% foetal bovine serum (FBS, Gibco, Carlsbad, CA), 100 IU/mL penicillin/streptomycin at 37 °C, with 5% CO_2_ in a humidified incubator.

### CCK-8 cell viability assay

The indicated cells were seeded into a 96-well plate at a density of 5 × 10^3^ cells/well. IAL was dissolved in dimethyl sulphoxide (DMSO) and diluted with DMEM medium to indicated final concentrations. After incubation with IAL for indicated time, cell viability was detected using a CCK-8 assay (cat. no. 40203; Yeasen, Shanghai, China) according to the manufacturer’s instructions. After 1 h of incubation, the absorbance of solution at 450 nm was measured by a VersaMax microplate reader (Thermo, Waltham, MA).

### Colony-formation assay

NOZ and GBC-SD cells were seeded into a six-well plate at a density of 1 × 10^3^ cells/well and treated with IAL or Ro 67-7476 at indicated concentrations for 24 h. Then, the medium was changed with fresh medium and the cells were cultured for 12 days until they grew to visible colonies. The cells were washed with PBS and fixed with 4% paraformaldehyde for 20 min. Then, 0.1% crystal violet solution was used for staining and a full visual was captured by a gel imager (Bio-Rad, Hercules, CA).

### 5-Ethynyl-2′-deoxyuridine (EdU) staining assay

The indicated cells were seeded into 12-well plates at a density of 1 × 10^5^ cells/well. After the cells were incubated with 20 μM EdU working solution for 2 h, EdU staining was performed using an EdU Imaging Test Kit (Beyotime, Shanghai, China). Briefly, cells were fixed with 4% paraformaldehyde for 15 min, and then permeated with 0.3% TritonX-100 for 15 min and then incubated with staining reaction cocktail for 30 min in the dark. The nuclei were stained with Hoechst. All images were acquired using a fluorescence microscope (Eclipse E600; Nikon, Japan).

### Western blot

Western blot was performed according to a previous report (Lv et al. 2022). Briefly, cells and tumour tissues were homogenized on ice in RIPA lysis buffer (FUDE, Hangzhou, China) supplemented with protease and phosphatase inhibitors (MCE). Protein concentration was quantified using a Pierce bicinchoninic acid (BCA) protein assay kit (Thermo, Waltham, MA). Equal amounts of proteins were resolved by sodium dodecyl sulphate polyacrylamide gel electrophoresis (SDS-PAGE) and transferred onto PVDF membranes (Bio-Rad, Hercules, CA). The membranes were blocked in 5% skimmed milk for 1 h at room temperature and then incubated with the indicated primary antibodies at 4 °C overnight, and incubated with secondary peroxidase-conjugated antibodies for 1 h. Protein bands were visualized using the electrogenerated chemiluminescence reaction. ImageJ software (Bethesda, MD) was used for densitometric analysis. The antibodies used for western blot are displayed in Table 1.

**Table 1.** Antibodies used in western blot.

Antibody	Source/catalogue number	Dilution
cyclinD1	Cell Signaling Technology (Boston, MA)/55506	1:1000
CDK4	Cell Signaling Technology (Boston, MA)/12790	1:1000
N-cadherin	Abcam (Cambridge, UK)/ab76011	1:1000
Vimentin	Cell Signaling Technology (Boston, MA)/5741	1:1000
E-cadherin	Abcam (Cambridge, UK)/ab40772	1:1000
Bcl-2	Abcam (Cambridge, UK)/ab32124	1:1000
Bax	Abcam (Cambridge, UK)/ab32503	1:1000
Phospho-ERK (Thr202/Tyr204)	Cell Signaling Technology (Boston, MA)/4370	1:1000
ERK	Cell Signaling Technology (Boston, MA)/4696	1:1000
Phospho-JNK (Thr183/Tyr185)	Cell Signaling Technology (Boston, MA)/9255	1:1000
JNK	Cell Signaling Technology (Boston, MA)/9252	1:1000
Phospho-p38 (Thr180/Tyr182)	Cell Signaling Technology (Boston, MA)/4511	1:1000
p38	Cell Signaling Technology (Boston, MA)/8690	1:1000
Phospho-MEK (Ser221)	Cell Signaling Technology (Boston, MA)/2338	1:1000
MEK	Cell Signaling Technology (Boston, MA)/4694	1:1000
GAPDH	FUDE (Hangzhou, China)/FD0063	1:5000

### Wound healing assay

The NOZ and GBC-SD cells were seeded in the ibidi Culture-Insert (ibidi GmbH, Gräfelfing, Germany) at 4 × 10^4^ per Culture-Insert on 12-well plates. After appropriate cell attachment, the Culture-Insert was gently removed, and photomicrographs were taken under a microscope (Zeiss, Oberkochen, Germany) at 0 h. Next, each cell line was divided into 3–4 groups and treated as colony-formation assay mentioned previously. After wounding for 48 h, each wound was captured by a microscope (Zeiss, Oberkochen, Germany). All experiments were performed in triplicate.

### Transwell migration and invasion assay

The migration and matrigel invasion assays were conducted using transwell chamber (for migration assay) or transwell precoated matrigel chamber (for invasion assay) according to the manufacturer’s protocol (BD Biosciences, Franklin Lakes, NJ). The cells were treated with IAL or Ro 67-7476 for 24 h. Then, the homogeneous single-cell suspensions (4 × 10^4^ cells/well for migration, 8 × 10^4^ cells/well for invasion) were added to the upper chambers and incubated for 24 h. The numbers of migratory and invasive cells were quantified by counting at least three random fields.

### Flow cytometry

The NOZ and GBC-SD cells were seeded into a six-well plate at a density of 3 × 10^5^ cells/well. Then, the cells were treated with IAL or Ro 67-7476 for 24 h. For cell cycle assay, the cells were harvested, washed twice with PBS and stained with 1 mL DNA staining solution and 10 μL permeabilization solution for 30 min in the dark. For cell apoptosis assay, an Annexin V-FITC and propidium iodide (PI) kit (Multisciences, Beijing, China) was used according to the manufacturer’s instructions. Experiments were performed using a FACS Fortessa (BD Biosciences, Franklin Lakes, NJ) and the results were analysed with FlowJo software (TreeStar, Ashland, OR).

### Immunohistochemistry and H&E staining

The tissues were fixed overnight in the formalin solution, dehydrated in a graded alcohol series and embedded in paraffin. Paraffin-embedded samples were cut at 5 μm thickness. Immunohistochemical staining for Ki-67 (ab16667, Abcam, Cambridge, UK, 1:200) was performed using a DAB detection kit (GK600710, Gene Company, Shanghai, China) according to the manufacturer’s instructions. To measure Ki-67 positive cells, five random 200× fields were calculated per slide from six mice of each group. The results are represented as means, with error bars indicating the standard deviations (S.D.). For H&E staining, the tissues were stained with haematoxylin and eosin.

### Xenograft models

Eighteen female BALB/c nude mice (4 weeks old, 16–18 g) were purchased from ZiYuan Experimental Animals Company (Hangzhou, China). Mice were maintained under standard animal care conditions with free access to water and standard chow. To generate the NOZ cells-derived xenograft animal models, about 5 × 10^6^ NOZ cells in 80 μL PBS were subcutaneously injected into the axilla of each nude mouse. When the tumours in the dorsal area were macroscopic (day 7), nude mice were randomly divided into three groups (control group, IAL group and IAL + Ro 67-7476 group) with six mice in each group. For the control group, a volume of 100 μL 5% DMSO was injected into the peritoneum. The IAL group was received intraperitoneal injection of 100 μL IAL (10 mg/kg) diluted with 5% DMSO (Chen et al. 2018). The IAL + Ro 67-7476 group was received intraperitoneal injection of 100 μL IAL + Ro 67-7476 (IAL, 10 mg/kg; Ro 67-7476, 4 mg/kg) diluted with 5% DMSO (Bariselli et al. 2016). Since day 7, the intraperitoneal injection was done every day and the body weight and tumour volume of mice were measured every three days. The volume of tumour was calculated as the formula: tumour volume (mm^3^) = 0.5 × length (mm)×width (mm)^2^. Thirty days later, the nude mice were sacrificed and the tumours were harvested, photographed and weighed. The tumour samples were stored at −80 °C or preserved in 4% paraformaldehyde for further analysis. All animal experiments were approved by the Committee for Animal Experiments in Zhejiang University.

### Statistical analysis

Data presented in this study are representative of at least three independent experiments and expressed as means ± S.D. Statistical analysis was performed with GraphPad Prism software (version 8.0, La Jolla, CA). One-way ANOVA followed by Tukey’s *post hoc* test was used when comparing more than two groups of data. *p*< 0.05 was considered significant in statistics.

## Results

### Isoalantolactone suppresses the viability of the human gallbladder carcinoma cells *in vitro*

**Figure 1.**
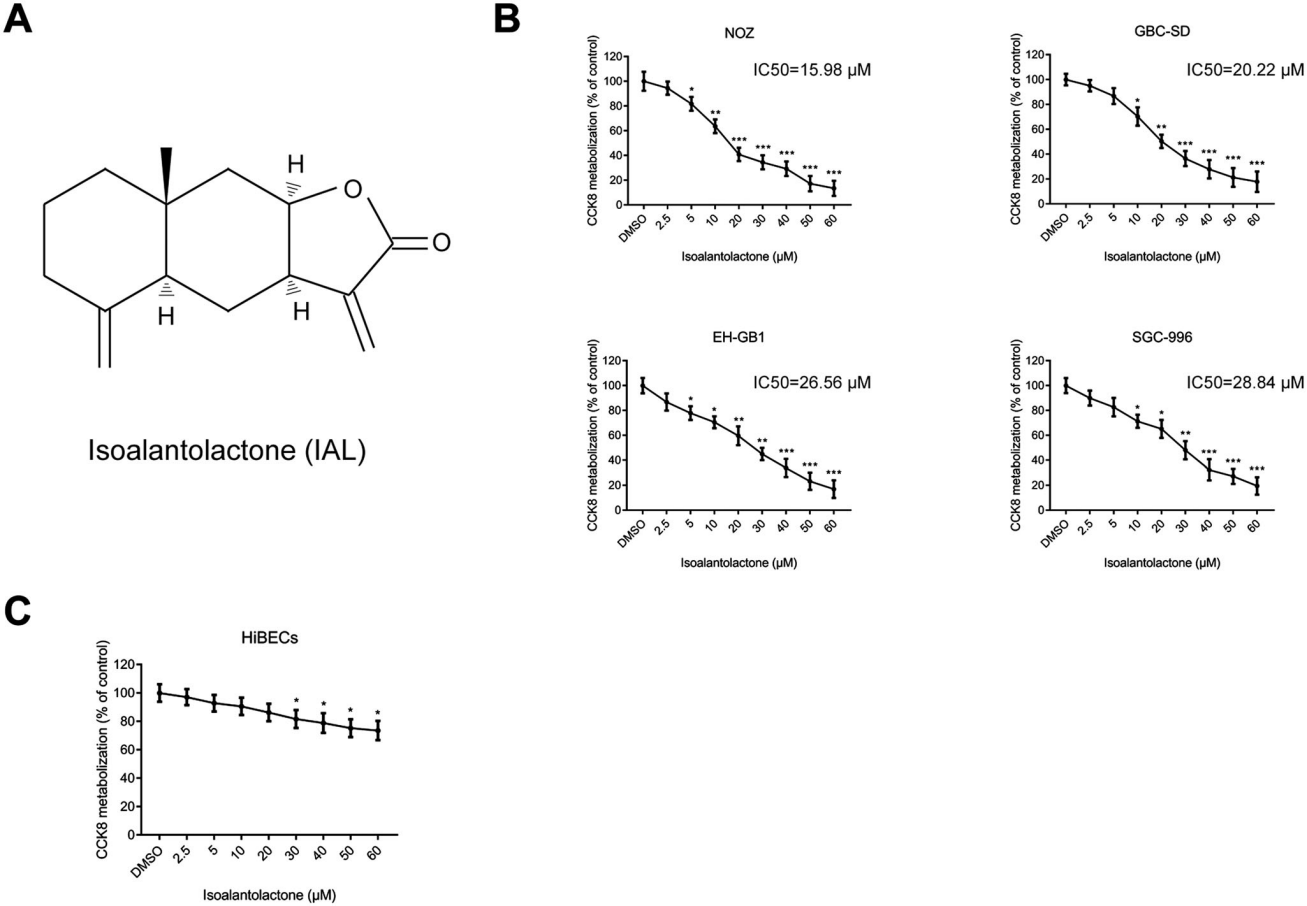
Isoalantolactone suppresses the viability of the human gallbladder carcinoma cells *in vitro*. (A) Chemical structure of isoalantolactone (IAL). (B) The anti-proliferative effects of IAL on NOZ, GBC-SD, EH-GB1 and SGC-996 cells were detected by CCK-8 assay (*n* = 3). (C) The anti-proliferative effects of IAL on HiBECs were detected by CCK-8 assay (*n* = 3). Cells were treated with different concentration of IAL for 24 h. Control group contained 0.1% DMSO. Data are mean ± S.D., *p< 0.05, **p< 0.01 and ***p< 0.001 compared to DMSO group.

The chemical structure of IAL is shown in Figure 1(A). To explore the effects of IAL on the growth of gallbladder carcinoma cells, four human gallbladder carcinoma cell lines, including NOZ, GBC-SD, EH-GB1 and SGC-996, were treated with IAL at various concentrations (0–60 μM) for 24 h. As shown in Figure 1(B), the CCK-8 assay results showed that IAL treatment decreased the viability of four cell lines. Moreover, the concentration to achieve 50% growth inhibition (IC_50_) of IAL in each GBC cell line was calculated to guide further research. The results showed that NOZ (IC_50_=15.98 μM) and GBC-SD (IC_50_=20.22 μM) cells were more sensitive to IAL treatment than EH-GB1 (IC_50_=26.56 μM) and SGC-996 (IC_50_=28.84 μM) cells. Therefore, the following analysis was conducted by using IAL at the concentration of 10, 20 and 40 μM in NOZ and GBC-SD cell lines. Besides, the normal HiBECs were also treated with IAL and the CCK-8 assay results showed that the growth inhibitory effect of IAL towards HiBECs was significantly lower compared to the GBC cells (Figure 1(C)). Taken together, these results suggested that IAL had highly selective cytotoxic effect on gallbladder carcinoma cells.

### Isoalantolactone inhibits the proliferation of NOZ and GBC-SD cells, resulting in *G*_0_/*G*_1_ phase arrest

**Figure 2.**
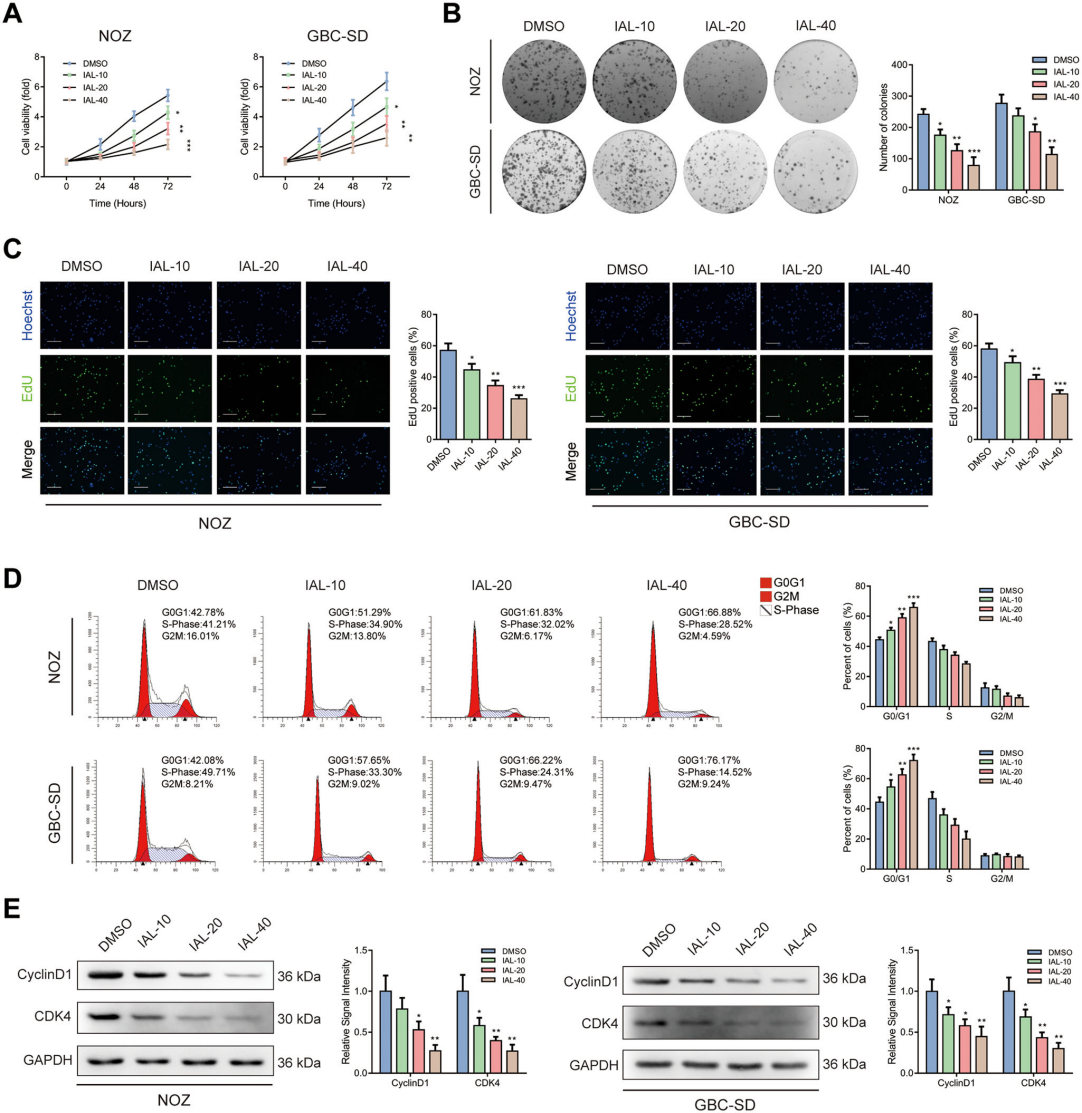
Isoalantolactone inhibits the proliferation of NOZ and GBC-SD cells, resulting in *G*_0_/*G*_1_ phase arrest. (A) NOZ and GBC-SD cells were treated with indicated concentrations of IAL for the indicated times, following which cell proliferation was analysed using the CCK-8 assay (*n* = 3). (B) The effects of IAL on the colony-formation ability in NOZ and GBC-SD cells as demonstrated by colony-formation assay (*n* = 3). (C) The effects of IAL on proliferation activity were measured by EdU staining in NOZ and GBC-SD cells. Representative photomicrographs and quantitative data of the percentages of EdU positive cells were shown (*n* = 3, scale bar = 100 µm). (D) Cell cycle assay of NOZ and GBC-SD cells treated with indicated concentrations of IAL (*n* = 3). (E) Protein levels of cyclinD1 and CDK4 in NOZ and GBC-SD cells treated with indicated concentrations of IAL. GAPDH served as loading controls (*n* = 3). Data are mean ± S.D., *p< 0.05, **p< 0.01 and ***p< 0.001 compared to DMSO group.

As shown in Figure 2(A), the CCK-8 assay indicated that cell proliferation of NOZ and GBC-SD cells was inhibited by IAL compared with the control group. Additionally, the colony formation assay displayed that IAL distinctly diminished the valid clones of NOZ and GBC-SD cells (Figure 2(B)). Moreover, we examined the effect on the proliferative capacity by EdU staining and found that IAL treatment decreased cell proliferation of NOZ and GBC-SD cells (Figure 2(C)). Besides, the results from the flow cytometry analysis of the effect of IAL treatment on cell cycle showed that IAL treatment significantly induced cell cycle *G*_0_/*G*_1_ arrest in NOZ and GBC-SD cells (Figure 2(D)). In addition, we performed western blot in NOZ and GBC-SD cells. The results showed that IAL treatment could suppress the expressions of cyclinD1 and CDK4 (Figure 2(E)). Taken together, these results indicated that IAL could inhibit cell proliferation and induce *G*_0_/*G*_1_ phase arrest in NOZ and GBC-SD cells.

### Isoalantolactone inhibits the migration and invasion of NOZ and GBC-SD cells and induces cell apoptosis

**Figure 3.**
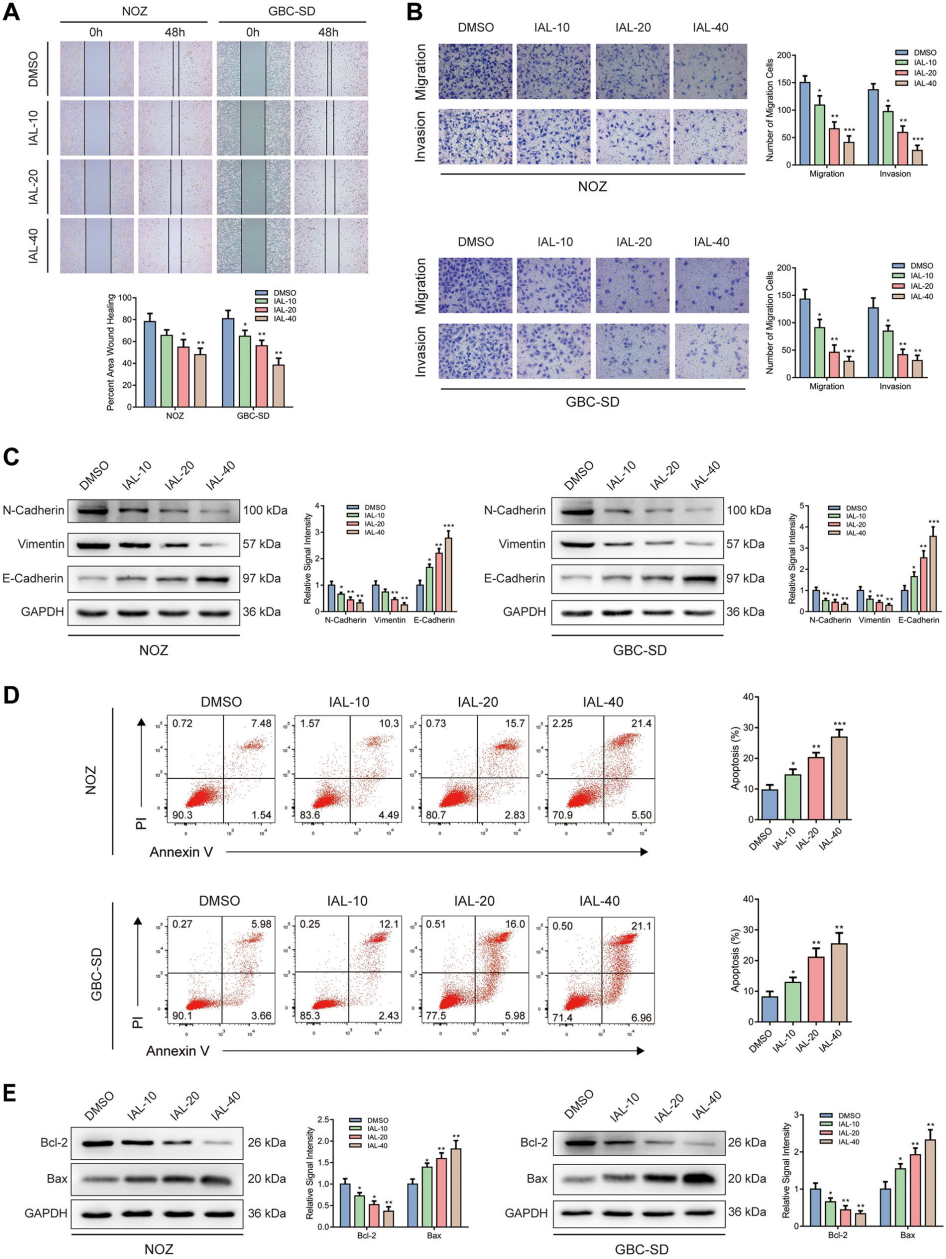
Isoalantolactone inhibits the migration and invasion of NOZ and GBC-SD cells and induces cell apoptosis. (A) Wound healing assay of NOZ and GBC-SD cells treated with indicated concentrations of IAL (*n* = 3). (B) Transwell migration and invasion assay of NOZ and GBC-SD cells treated with indicated concentrations of IAL (*n* = 3). (C) Protein levels of N-cadherin, vimentin and E-cadherin in NOZ and GBC-SD cells treated with indicated concentrations of IAL. GAPDH served as loading controls (*n* = 3). (D) Cell apoptosis assay of NOZ and GBC-SD cells treated with indicated concentrations of IAL (*n* = 3). (E) Protein levels of Bcl-2 and Bax in NOZ and GBC-SD cells treated with indicated concentrations of IAL. GAPDH served as loading controls (*n* = 3). Data are mean ± S.D., *p< 0.05, **p< 0.01 and ***p< 0.001 compared to DMSO group.

Interestingly, a comparison of control and IAL groups in wound healing assay showed that the healing rates of NOZ and GBC-SD cells were distinctly restrained by IAL incubation (Figure 3(A)). Similar results were acquired by transwell migration and invasion assay. The migration and invasion of NOZ and GBC-SD cells were suppressed by IAL treatment (Figure 3(B)). Moreover, western blot analysis suggested that the expressions of epithelial–mesenchymal transition (EMT) markers, including N-cadherin and vimentin, were significantly down-regulated and the expression of E-cadherin was significantly up-regulated after IAL treatment (Figure 3(C)). In addition, we also performed Annexin V/PI double staining to identify the cell apoptosis in NOZ and GBC-SD cells by flow cytometry. Compared with the control group, the proportion of cell apoptosis was effectively increased by IAL treatment (Figure 3(D)). Besides, as shown in Figure 3(E), IAL treatment increased the expression of pro-apoptotic protein Bax and decreased the expression of anti-apoptotic protein Bcl-2, indicating that IAL perturbed the key proteins involved in intrinsic apoptosis. Taken together, these results indicated that IAL inhibited the migration and invasion of NOZ and GBC-SD cells and induced cell apoptosis.

### Isoalantolactone inhibits the ERK signalling pathway and Ro 67-7476 offsets the IAL-related proliferation inhibition

**Figure 4.**
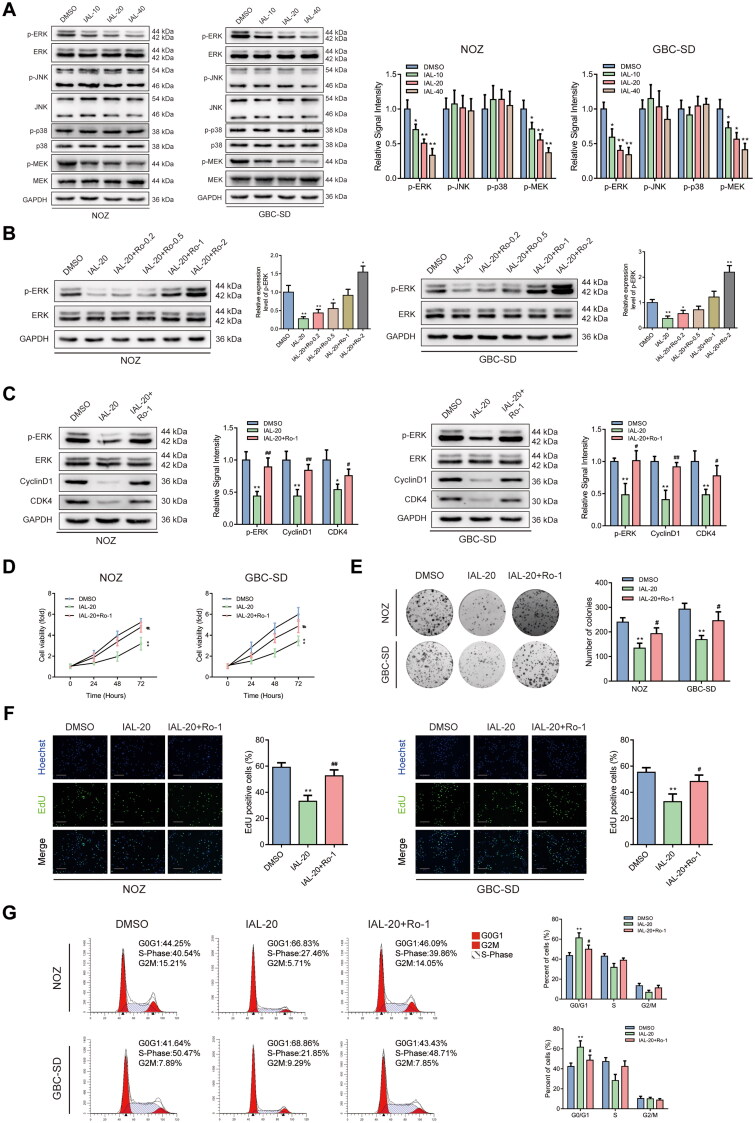
Isoalantolactone inhibits the ERK signalling pathway and Ro 67-7476 offsets the IAL-related proliferation inhibition. (A) Protein levels of p-ERK, ERK, p-JNK, JNK, p-p38, p38, p-MEK and MEK in NOZ and GBC-SD cells treated with indicated concentrations of IAL. GAPDH served as loading controls (*n* = 3). (B) Protein levels of p-ERK and ERK in NOZ and GBC-SD cells treated with IAL and indicated concentrations of Ro 67-7476. GAPDH served as loading controls (*n* = 3). (C) Protein levels of p-ERK, ERK, cyclinD1 and CDK4 in NOZ and GBC-SD cells treated with IAL or Ro 67-7476. GAPDH served as loading controls (*n* = 3). (D) NOZ and GBC-SD cells were treated with IAL or Ro 67-7476 for the indicated times, following which cell proliferation was analysed using the CCK-8 assay (*n* = 3). (E) The effects of IAL and Ro 67-7476 on the colony-formation ability in NOZ and GBC-SD cells as demonstrated by colony-formation assay (*n* = 3). (F) The effects of IAL and Ro 67-7476 on proliferation activity were measured by EdU staining in NOZ and GBC-SD cells. Representative photomicrographs and quantitative data of the percentages of EdU positive cells were shown (*n* = 3, scale bar = 100 µm). (G) Cell cycle assay of NOZ and GBC-SD cells treated with IAL and Ro 67-7476 (*n* = 3). Data are mean ± S.D., *p< 0.05 and **p< 0.01 compared to DMSO group, ^#^*p*< 0.05 and ^##^*p*< 0.01 compared to IAL-20 group.

The mitogen-activated protein kinases (MAPKs) pathway is involved in multiple cellular processes, including proliferation, migration, invasion and apoptosis (Kim and Choi 2015). Previous evidences indicated that IAL could regulate the MAPK signalling pathway in human breast cancer (Li et al. 2016; Wang et al. 2016). Therefore, we speculated that IAL could inhibit the proliferation, migration and invasion and induced cell apoptosis of GBC cells through the MAPK signalling pathway. To verify this hypothesis, we detected the phosphorylation levels of ERK, JNK and p38 to evaluate the MAPK signalling pathways by western blot analysis. The phosphorylation level of ERK was remarkably decreased after IAL treatment, while the expression of total ERK had no change. However, the activation of JNK and p38, which were known as other members of the MAPK family, was barely affected by IAL treatment. Moreover, we detected the phosphorylation level of MEK by western blot analysis. The results showed the phosphorylation level of MEK was remarkably decreased after IAL treatment, while the expression of total MEK had no change, which indicates that IAL inhibited the MEK/ERK signalling pathway (Figure 4(A)). Then, Ro 67-7476, the ERK phosphorylation agonist, was introduced in this study. We confirmed that Ro 67-7476 could up-regulate p-ERK in NOZ and GBC-SD cells as demonstrated by western blot analysis and chose 1 μM of Ro 67-7476 in the following experiments (Figure 4(B)). Interestingly, western blot analysis showed that the effects of IAL on the protein levels of p-ERK, cyclinD1 and CDK4 were reversed by Ro 67-7476 (Figure 4(C)). Moreover, the CCK-8 assay suggested that Ro 67-7476 offsets the IAL-related cell proliferation inhibition in NOZ and GBC-SD cells (Figure 4(D)). Besides, the images displayed in Figure 4(E) declared that Ro 67-7476 was capable of reversing adverse effect from IAL on valid clone formation in NOZ and GBC-SD cells. Furthermore, the effects of IAL on proliferative capacity were also reversed by Ro 67-7476 in the EdU staining assay (Figure 4(F)). In addition, as shown in Figure 4(G), IAL led to *G*_0_/*G*_1_ cell cycle arrest in NOZ and GBC-SD cells, and the arrest effect was cancelled after Ro 67-7476 incubation. Taken together, these results indicated that IAL inhibited the ERK signalling pathway and Ro 67-7476 offsets the IAL-related proliferation inhibition.

### Ro 67-7476 antagonizes the IAL-related migration and invasion inhibition and the IAL-related cell apoptosis promotion

**Figure 5.**
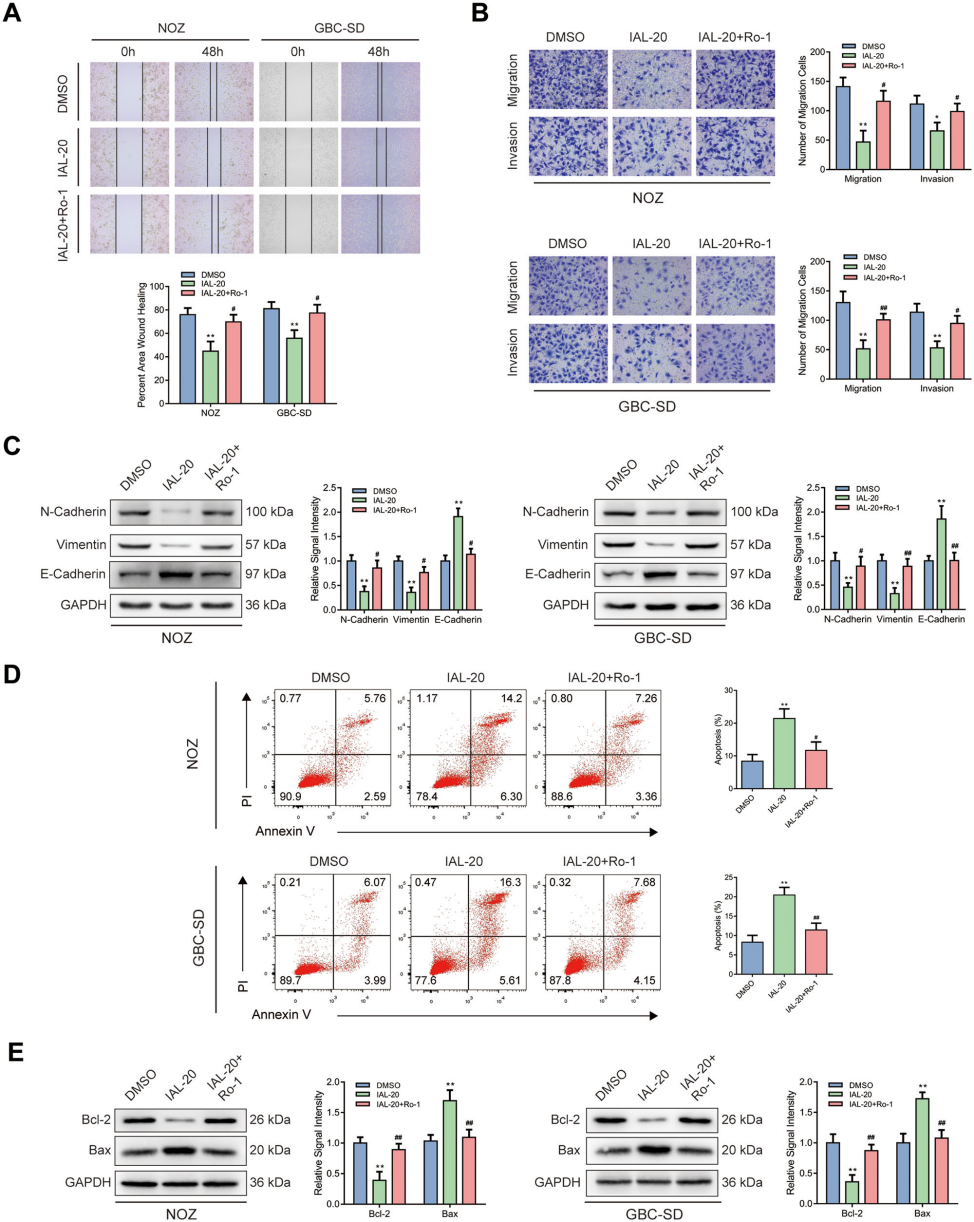
Ro 67-7476 antagonizes the IAL-related migration and invasion inhibition and the IAL-related cell apoptosis promotion. (A) The effects of IAL and Ro 67-7476 on the migration ability in NOZ and GBC-SD cells as demonstrated by wound healing assay (*n* = 3). (B) Transwell migration and invasion assay demonstrated the effects of IAL and Ro 67-7476 on the migration and invasion ability in NOZ and GBC-SD cells (*n* = 3). (C) Protein levels of N-cadherin, vimentin and E-cadherin in NOZ and GBC-SD cells treated with IAL or Ro 67-7476. GAPDH served as loading controls (*n* = 3). (D) Cell apoptosis assay of NOZ and GBC-SD cells treated with IAL and Ro 67-7476 (*n* = 3). (E) Protein levels of Bcl-2 and Bax in NOZ and GBC-SD cells treated with IAL or Ro 67-7476. GAPDH served as loading controls (*n* = 3). Data are mean ± S.D., *p< 0.05 and **p< 0.01 compared to DMSO group, ^#^*p*< 0.05 and ^##^*p*< 0.01 compared to IAL-20 group.

Notably, assessed by wound healing assay, wound healing rates of NOZ and GBC-SD cells impaired by IAL were nearly repaired after Ro 67-7476 incubation (Figure 5(A)). Moreover, the effects of IAL on cell migration and invasion were also reversed by Ro 67-7476 in transwell migration and invasion assay (Figure 5(B)). Similar results were acquired by western blot. Ro 67-7476 possessed antagonism against the effect that IAL down-regulated the protein levels of N-cadherin and vimentin and up-regulated the level of E-cadherin in NOZ and GBC-SD cells (Figure 5(C)). Besides, flow cytometry was utilized to analyse cell apoptosis of NOZ and GBC-SD cells. As expected, compared with the control group, the apoptosis ratios of NOZ and GBC-SD cells were augmented by IAL but diminished by Ro 67-7476. Cell apoptosis was rescued in the IAL-20 + Ro-1 group compared with IAL-20 group in NOZ and GBC-SD cells (Figure 5(D)). In addition, Ro 67-7476 possessed antagonism against the effect that IAL down-regulated the protein level of Bcl-2 and up-regulated the level of Bax in NOZ and GBC-SD cells (Figure 5(E)). Taken together, these results suggested that Ro 67-7476 antagonized the IAL-related migration and invasion inhibition and the IAL-related cell apoptosis promotion.

### Isoalantolactone inhibits NOZ xenograft tumour growth via the ERK signalling pathway *in vivo*

**Figure 6.**
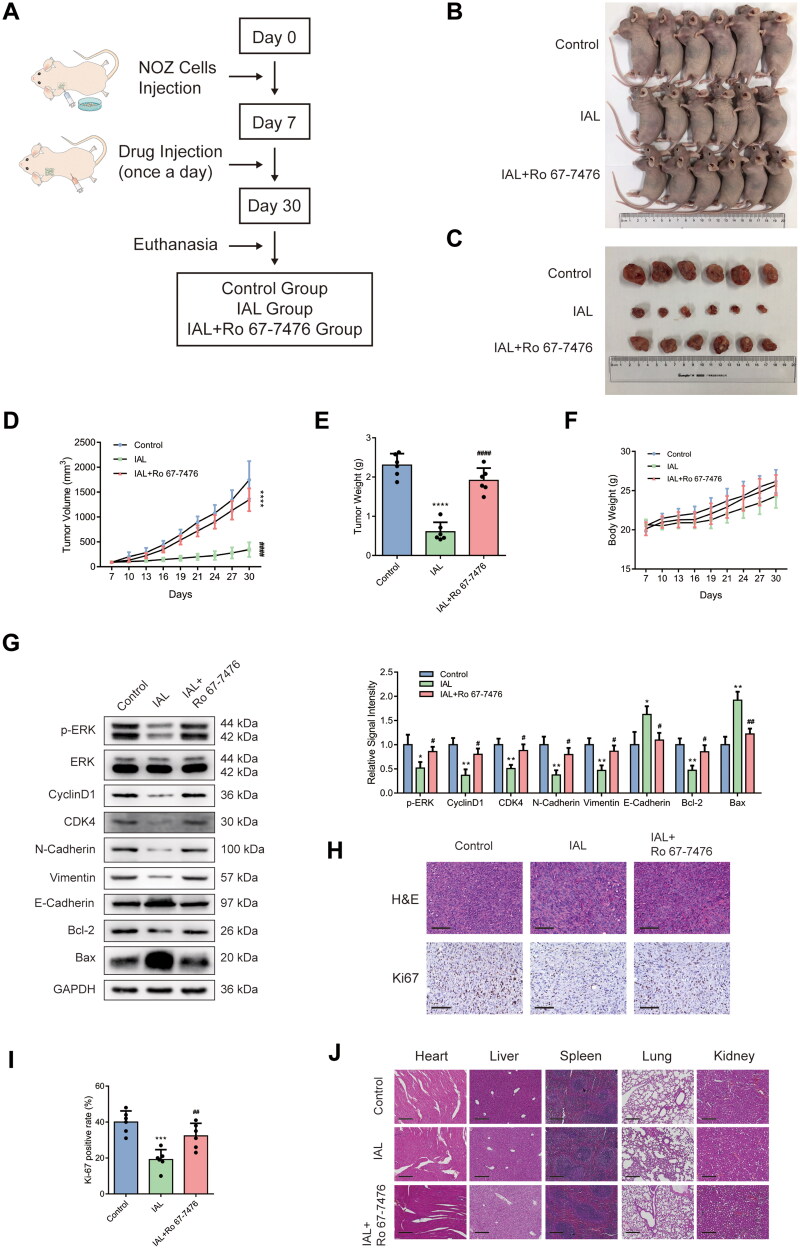
Isoalantolactone inhibits NOZ xenograft tumour growth via the ERK signalling pathway *in vivo*. (A) Establishment and treatment of a tumour-transplanted mouse model using NOZ cells. (B) Tumour-transplanted nude mice images after 30 days in different groups (*n* = 6 mice). (C) Tumour images after 30 days in different groups (*n* = 6 mice). (D) Tumour volume changes in different groups (*n* = 6 mice). (E) Statistical plot of the average tumour weights of the different groups (*n* = 6 mice). (F) Nude mice body weight changes in different groups (*n* = 6 mice). (G) Protein levels of p-ERK, ERK, cyclinD1, CDK4, N-cadherin, vimentin, E-cadherin, Bcl-2 and Bax in tumours were evaluated by western blot analysis. GAPDH served as loading controls (*n* = 6 mice). (H) Representative images of H&E and Ki-67 staining of collected tumour sections from different groups at 30 days (scale bar = 100 µm). (I) Quantitation of Ki-67 positive cells from different groups at 30 days (*n* = 6 mice). (J) The histology of heart, liver, spleen, lung and kidney was evaluated by H&E staining (scale bar = 200 µm). Data are mean ± S.D., *p< 0.05, **p< 0.01, ***p< 0.001 and ****p< 0.0001 compared to control group, ^#^*p*< 0.05, ^##^*p*< 0.01 and ^####^*p*< 0.0001 compared to IAL group.

A tumour-transplanted mouse model was introduced in this study to investigate the effect of IAL on tumour growth *in vivo*. Nude mice were randomly divided into three groups (control group, IAL group and IAL + Ro 67-7476 group) (Figure 6(A)). As shown in Figure 6(B–E), the results demonstrated that intraperitoneal injection of IAL at dose of 10 mg/kg markedly suppressed NOZ cell-derived xenograft tumour growth in terms of volume and weight, while Ro 67-7476 could reverse the antitumour effect of IAL. Besides, there was no significant difference of body weight among the three groups (Figure 6(F)). To determine whether the *in vitro* mechanisms also play a role in the *in vivo* studies, a series of western blot were conducted to assess protein levels of tumour tissues. As shown in Figure 6(G), western blot results showed that IAL treatment led to a decrease in the protein levels of p-ERK, cyclinD1, CDK4, N-cadherin, vimentin and Bcl-2 and an increase in the levels of E-cadherin and Bax, while Ro 67-7476 could also reverse the effect of IAL. Moreover, the change in morphology was evaluated by H&E staining, which showed the abnormality of cell shape (Figure 6(H)). Meanwhile, the expression of Ki-67 detected by immunohistochemistry staining was obviously diminished in IAL group, while Ro 67-7476 could also reverse the effect of IAL (Figure 6(H,I)). In addition, the major organs, including heart, liver, spleen, lung and kidney, were collected to investigate the potential cytotoxic effect of IAL on normal tissues. The H&E staining results revealed that IAL and Ro 67-7476 treatment did not result in observable toxicity (Figure 6(J)). Taken together, these results suggested that IAL inhibited NOZ xenograft tumour growth *in vivo* via inhibiting the ERK signalling pathway, which was in accordance with the mechanism *in vitro*.

## Discussion

Gallbladder cancer usually originates in the epithelial cells of the biliary duct system (Shen et al. 2019; Mao et al. 2020; Chen et al. 2021). According to the Surveillance, Epidemiology, and End Results (SEER) program database, GBC has a poor prognosis, with a median survival time of less than 12 months and a 5-year overall survival (OS) rate of less than 5% (Hundal and Shaffer 2014; Cai et al. 2020; Mao et al. 2020). Surgery is currently the only treatment option, but most patients are diagnosed too late for surgery, as GBC onset and progression are usually asymptomatic at early stage (Roessler et al. 2021). If GBC has metastases at the time of diagnosis, surgery is usually not indicated and chemotherapy is the first choice of treatment (Baiu and Visser 2018). Although gemcitabine-based systemic chemotherapy offers a treatment option, only a few patients yield promising prognoses due to severe systemic toxicity and drug resistance (Azizi et al. 2021). Therefore, novel potential antitumour drugs need to be explored to improve the quality of life and OS of GBC patients.

Previous studies have shown that IAL, the root extract of *Inula helenium*, has the activity of inhibiting various cancers. Likewise, Kim et al. (2021) demonstrated that IAL suppressed HCC cells via the JNK signalling pathway; Li et al. (2016) suggested that IAL induced apoptosis in human breast cancer cells via ROS-mediated mitochondrial pathway; Lu et al. (2018) indicated that IAL induced apoptosis in human oesophageal cancer cells; Chen et al. (2018) found that IAL induced apoptosis through inhibition of STAT3 in prostate cancer cells; Weng et al. (2016) indicated that IAL induced autophagic cell death in human ovarian carcinoma cells via up-regulation of PEA-15; Khan et al. (2012) indicated that IAL inhibited pancreatic carcinoma progression. Uncontrolled growth and inhibition of apoptosis help cancer cells to grow rapidly. Therefore, anticancer drugs usually work by inhibiting cell proliferation and promoting apoptosis. In this study, we found that IAL induced *G*_0_/*G*_1_ phase arrest and induced apoptosis through cyclinD1 and CDK4, which are key regulatory factors regulating *G*_0_/*G*_1_ transition.

Epithelial–mesenchymal transition is a cellular process in which epithelial cells lose apical–basolateral polarity, disassemble cell–cell junctions and become progressively motile, and it plays an important role in the invasion and metastasis of cancer (Dongre and Weinberg 2019). Vimentin is an important component of the intermediate filament protein family and a biomarker for predicting tumour metastasis, which is closely related to the invasion and metastasis of various malignant tumours (Satelli and Li 2011; Al-Maghrabi 2020). N-cadherin, a calcium-dependent single-chain transmembrane glycoprotein, is another key EMT marker that mediates homotypic and heterotypic cell–cell adhesion (Cao et al. 2019). The above proteins play an important role in the evolution of EMT and our study also verified the characteristic changes of these proteins. The results showed that IAL inhibited the migration and invasion of GBC cells by inhibiting the protein levels of the EMT-related factors N-cadherin and vimentin.

The MAPK signalling pathway is a key intracellular signal transduction that regulates a variety of cellular activities and is commonly dysfunctional in human malignancies (Cubero et al. 2020; Asl et al. 2021). The MAPKs include extracellular signal-regulated kinase (ERK), p38 MAPK and c-Jun NH_2_-terminal kinase (JNK). The p38 MAPK and JNK signalling pathways are activated by various types of cellular stress, such as osmotic and oxidative stress as well as pro-inflammatory cytokines such as interleukin 1β (IL1β) and tumour necrosis factor-α (TNFα). The Ras/Raf/MEK/ERK signalling pathway plays a key role in cancer development by stimulating cell metastasis and proliferation (Kim and Choi 2015; Cingolani et al. 2022). When MEK1/2 activated by RAFs induces phosphorylation of ERK1 or ERK2, p-ERK1 or p-ERK2 is dimerized and translocated to the nucleus, where transcription factors are activated to regulate gene expression (Khokhlatchev et al. 1998). Previous studies have shown that some traditional Chinese medicines, such as bufalin, pachymic acid and artemisinin, could inhibit GBC cell proliferation and metastasis by interrupting the ERK signalling pathway (Chen et al. 2015; Wang et al. 2016; Qian et al. 2020). In this study, we detected the phosphorylation levels of ERK, JNK, p38 and MEK to evaluate the MAPK signalling pathways. The ERK and MEK phosphorylation levels were remarkably decreased after IAL treatment in NOZ and GBC-SD cells, while the expression of total ERK and MEK had no change. Nevertheless, the activation of JNK and p38, which were known as other members of the MAPK family, was hardly affected by IAL treatment. In addition, Ro 67-7476, the ERK phosphorylation agonist, neutralized IAL-induced antitumour effects *in vitro* and *in vivo*. These results revealed that MEK/ERK signalling pathway was an important mediator of IAL in GBC.

The ERK signalling pathway is required for EMT and influence invasion at several levels. First, the ERK signalling pathway cooperates with other pathways (mainly, the TGFβ–Smad pathway) to upregulate expressions of EMT-related genes, including mesenchymal genes and transcription repressors of epithelial genes such as snail, slug, Twist1/2 and ZEB1/2 (Lehmann et al. 2000; Tan et al. 2004; Sobczak et al. 2008; Riesco-Eizaguirre et al. 2009). Second, the ERK pathway plays an essential role in cell contractility and migration by regulating the Rho/Rac-actin pathway (Pritchard et al. 2004; Ehrenreiter et al. 2005). Besides, increased expression of matrix metalloproteinases by the ERK pathway led to a remodelling of the microenvironment, which induces loss of tissue polarity and reinitiation of proliferation (Beliveau et al. 2010).

In this study, IAL showed a strong growth inhibitory effect towards NOZ and GBC-SD cells. However, this growth inhibitory effect of IAL towards HiBECs was significantly lower compared to GBC cells. This selective cytotoxicity may be due to much lower level of the ERK signalling pathway in normal cells than cancer cells. However, detailed investigations of this selective cytotoxicity of IAL still need to be performed.

## Conclusions

Our results suggest that IAL significantly suppresses GBC progression both *in vitro* and *in vivo* by inhibiting the ERK signalling pathway. Besides, IAL poses little toxicity *in vivo*. Therefore, our study provides a theoretical basis for the clinical application of IAL in the treatment of GBC.
